# The Canadian Medical Association

**Published:** 1889-10

**Authors:** 


THE CANADIAN MEDICAL ASSOCIATION held its twenty-second annual meeting at Banff, N. W. T., Aug. 12 and 13, 1889. A number of guests were present from the United States, and the meeting appears to have been a very successful one.

